# Medical Sanity

**Published:** 1891-05-30

**Authors:** 


### THE DOCTOR'S ARMCHAIR.

### MEDICAL SANITY.

SANITY is the root of truth, of justice, and of all right living. The honest practice of medicine tends eminently in the direction of sanity. It is hardly possible for a doctor who practises his art intelligently and sincerely to be a fool. Such a doctor may not necessarily be a clever man, but he is bound to be a sober one. He may, as well as another, be endowed with a soaring and wide-ranging imagination, but he can never be lost in unknown regions, because he is always held in daily check by the rein of actual, objective, material fact. To him will, purpose, and even the most commanding genius count for so very little under many of the circumstances of his life that he always feels the limits of the possible not only to be almost within sight on every side, but to be quite close at hand.

Is it a good thing for any considerable number of educated men to be made so convincedly conscious of the bars of the cage in which they live and work? Is it well to have too urgent a sense of one's own imprisonment and comparative impotence? The poet who is thought to be so much at the mercy of his own genius, and the preacher who may feel himself carried to immeasurable heights by some inexplicable inspiration—have not they the advantage of the practitioner of the medical art who knows always what to expect, and who is under the daily necessity of bounding his expectations within extremely moderate limits? A just judgment and a penetrating philosophy would probably decide in favour of the doctor. If anything could justify unchecked flights into the unknown it would be the feeling and impulsion of a divine inspiration. At all times men have allowed much freedom to him who has claimed the rights of prophet. But even here, in this unfamiliar region, sensitively honest souls have recognised limitations. St. Paul, a man of true science and sanity, lays it down as an axiom for the guidance of men claiming divine inspiration that "the spirits of the prophets are subject to the prophets."

Medicine will not permit a man to ride a winged-horse out of sight into the measureless sky for more than a very brief period. She soon brings him down to earth again. A patient in a typhoid fever cannot have his temperature lowered and kept steadily low by a mere theory of antipyretics however brilliant; an unhappy consumptive continues to cough and to lose flesh and strength in spite of all the scientific and logical forces which should theoretically destroy the tubercle bacillus, and give the disintegrating lung opportunity and encouragement to heal; old age insists upon its stealthy but sure approach, and no skill discoverable by the most strenuous genius of man can prevent it, with its companion, death, from finally claiming its own. If any man feels the iron heel of necessity standing upon his neck in his daily work, it is the doctor.

The kind of steadfast sanity, which is inevitable in the ablest medical men, is much needed as ballast in a state of society like our own. Every one of us knows how to value a solid and well-built house for living in. We should feel no pleasure in hearing the roof creak and shiver, or in feeling the walls and the floors swaying backwards and forwards with every winter storm. The consciousness of a rocky foundation, of solidly-constructed walls, and of sound beams that bind all the parts of the house together, is a distinct satisfaction in wild weather. But it is better to live in a rickety house than in a rickety state of society. Our fellow men, if unrestrained by law, custom, religion, and other such controlling forces, could do us a thousand times more harm by indiscriminately falling upon us and our belongings, than a tottering house could do us by its collapse and wreck. A view of things a little deeper than the everyday glance, soon shows us the uncomputable value of principles, science, and institutions that make for fixedness, sanity, order, and security in our dealings with our too little instructed fellows; that is, in the largest sense, in our state and social affairs.

We speak of the removal of old land-marks with light hearts. But such removal is always accompanied by risk; and there are two factors which markedly increase the risk; the first is the too great rapidity in deciding upon the removal of such land-marks; and the second is the large number of persons who insist upon their removal, as compared with the small number who may wish the old landmarks to remain. In other words, a rapidly growing population with its neccessary accompaniment of a fluid and mobile state of general ideas, is a distinctly dangerous condition of affairs. It is a condition of affairs in which bed rock foundations, firm barriers of separation, strongly built boundary walls of religion, science, custom, and the like are of the greatest possible importance as means of safety and ordered progress.

It is exactly here where the peculiar sanity of the doctor and the physiologist shows its exceeding value. Religious ideas and institutions, we know, change and often for the better. Political and social usages also change, and, perhaps, mostly for the better. But the facts of physiology, and to a large extent, those of disease, do not change. The physical man in his essence, and in most of his accidents, is the same as he always has been. Here is a basis of permanence, a foundation which does not shift, a bed rock which can be absolutely relied upon to remain where it is and as it is. The philosophic doctor, whose mind is compelled to daily dealing and constant familiarity with this immovable bed rock, this essentially permanent set of facts, this little-changing series of accidents, gradually acquires a firm conviction and assurance of reality, permanence, and unchanging order. Such a firm assurance of reality and permanence is the indispensable condition of all human well-being. The doctor cannot help being sane, because he cannot help being restrained by law and fact, and yet, feeling free, within certain limits, to accomplish work which is great in itself, and, at the same time, immediately serviceable to those who are the subjects of it.

To the doctor appertains the rare privilege of being free to act both as a Conservative and as a Radical. Rational medicine is before all things conservative. It does not give even so much as a single rhubarb pill to disturb the physiological equilibrium unless it be necessary. On the other hand, it is sometimes so radical as to be revolutionary. There can be no greater revolution in a man's activities than the cutting off of both his legs to save his life. The sanity of medicine consists in this—that it does not live by watchwords or parties, but by the guidance of permanent facts and large principles, urged into special action by the indispensable necessities of the moment.

The permanence of the human constitution, and the limited measure of variability in its activities, clearly make for intellectual steadiness, sanity, and safety in those individuals, whose work consists in understanding and dealing with that constitution. The reasonable inference is that, if elements as permanent as the physical constitution of man can be found in religion, in morals, in social and State affairs, they will have a like effect in producing religious, social, and political sanity. There is no doubt that such permanent elements do exist, and that they are as obvious and familiar to the best minds as are the facts of human physiology to the best physicians. What is wanted in a large and rapidly-growing population is the rapidly extended knowledge of these permanent elements. For just as a few eminent and intelligent physiological physicians have entirely failed to keep the whole population abreast of the knowledge of the permanent elements of bodily health and sanity, so will the limitation of the knowledge of the permanent elements of religious, social, and political sanity to a few, leave the many in a state of dangerous intellectual fluidity—a fluidity which only needs the agitation of boisterous winds and waves to be turned into insanity. The moral of the story is that in every department of human activity those elements that are permanent should be sought and recognised, and that every living man should be taught to understand their value.

